# Sexual satisfaction and attitude toward marital infidelity among married people in Iran: the role of social media and entertainment preferences

**DOI:** 10.1186/s12889-024-19073-w

**Published:** 2024-06-25

**Authors:** Abouzar Nazari, Maede Hosseinnia, Elahe Najafi

**Affiliations:** 1 Department of Health Education and Promotion, School of Public Health, Tehran University of Medical Sciences, Tehran, Iran; 2 Department of Health Education and Promotion, School of Public Health, Isfahan University of Medical Sciences, Tehran, Iran; 3 Department of Health Policy, School of Public Health, Tehran University of Medical Sciences, Tehran, Iran

**Keywords:** Sexual satisfaction, Marital infidelity, Social media, Entertainment preferences, Iran

## Abstract

**Background and purpose:**

Sexual satisfaction and attitudes toward marital infidelity are crucial components of marital quality and well-being. This study investigates the impact of social media and entertainment preferences on these aspects among married couples in Iran, acknowledging the sociocultural nuances unique to the region.

**Method and material:**

A cross-sectional survey design was employed, gathering data from 1,756 married participants through an online questionnaire in Iran. Variables included social media and entertainment preferences, sexual satisfaction, attitude toward marital infidelity, and demographic details. Descriptive statistics, non-parametric tests (Mann-Whitney U test, Kruskal-Wallis test), and GLM (Generalized linear model) were used for exploration.

**Results:**

Key results reveal significant associations between demographic factors, social media usage, and attitudes toward marital infidelity. Notable trends include higher sexual satisfaction among younger participants (*p* < 0.05), those with shorter marital durations (*p* < 0.01), and those residing outside Tehran (*p* < 0.001). Attitudes toward marital infidelity were influenced by gender, age, ethnicity, income levels, and social media habits, reflecting a complex interplay of factors. The GLM analysis emphasizes the impact of variables such as marital duration, ethnicity, spouse’s education, Iranian social media usage, and attitudes toward marital infidelity on sexual satisfaction. Participants with shorter marriages (*p* < 0.01), higher spouse education (*p* < 0.05), and more frequent Iranian social media usage (*p* < 0.001) reported higher sexual satisfaction.

**Conclusions:**

This study explores the dynamics of marital relationships in Iran, examining the interconnections between demographics, media habits, sexual satisfaction, and attitudes toward infidelity. The findings provide valuable insights into factors influencing marital satisfaction, emphasizing the importance of considering cultural contexts. Robust statistical methods, including Generalized Linear Models, support the reliability of results. The study contributes to understanding non-western marital dynamics, highlighting implications for research and interventions in the digital age.

## Introduction

Sexual health and satisfaction are crucial components of overall well-being, exerting profound influences on individuals’ psychological, emotional, and relational dimensions [1, 2]. This study explores the intricate interplay between social media, entertainment preferences, and key dimensions of marital dynamics, namely sexual satisfaction and attitudes toward marital infidelity. Unlike prevailing research largely conducted in Western contexts, this investigation places emphasis on the distinct sociocultural landscape of Iran.

### Sexual satisfaction: a multifaceted phenomenon

Sexual satisfaction, denoting the subjective evaluation of one’s sexual activity or relationship, encompasses diverse factors spanning biological to sociocultural influences [3, 4]. Biological facets include age, gender, hormonal balance, physical health, and sexual function, while psychological factors encompass personality traits, mood, self-esteem, and sexual beliefs. The relational dimension involves communication skills, intimacy, attachment styles, and partner compatibility. Crucially, sociocultural factors such as religion, education, income, and media exposure contextualize sexual satisfaction [5, 6].

### Social Media and Entertainment preferences: unexplored realms

In the contemporary landscape, social media and entertainment preferences play pivotal roles in shaping individuals’ identities, values, and behaviors [7, 8]. However, limited research exists on how these preferences impact the intimate realm of sexual satisfaction among married couples. This study seeks to address this gap by exploring the choices individuals make in terms of social networking sites, movies, series, music, and other entertainment mediums.

### Marital infidelity: a Nuanced Perspective

Understanding attitudes toward marital infidelity is crucial for comprehending the dynamics of marital quality and stability. This dimension involves the degree to which individuals accept or approve of extramarital relationships, presenting significant implications for individual and public health [9, 10]. While prior studies have predominantly focused on individual and relational factors influencing this attitude, the role of sociocultural factors, including social media and entertainment preferences, remains underexplored, particularly in non-Western contexts [11, 12].

### Study purpose and objectives

This study, conducted in Iran, aims to unravel the intricate relationships between social media, entertainment preferences, sexual satisfaction, and attitudes toward marital infidelity among married couples. By employing a cross-sectional survey design with a diverse sample of 1,756 participants, we seek to bridge gaps in the existing literature and provide insights into the unique sociocultural influences at play [13, 14]. Through robust statistical analyses, including Generalized Linear Models (GLM), we intend to elucidate the associations and shed light on the nuanced patterns that may emerge.

In conclusion, this research endeavors to contribute to the understanding of sexual health within the context of marriage, emphasizing the pivotal roles played by social media and entertainment preferences. The subsequent sections detail the methodology, results, and discussions, offering a comprehensive exploration of the intricate dynamics influencing marital satisfaction and attitudes toward infidelity among couples in Iran.

## Method

### Study design and participants

The current research is a descriptive-analytical study that aimed to investigate the relationship between social media and entertainment preferences and marital infidelity among married Instagram users in Iran. The study was conducted in two separate periods: from June 15, 2021, to July 16, 2021, and from May 20, 2023, to June 24, 2023. It adhered to the ethical guidelines of the American Psychological Association (APA) and received approval from the Institutional Review Board (IRB) of the Isfahan University of Medical Sciences. Participants were provided with information about the study’s purpose and procedures and were required to sign an online consent form before completing the questionnaire.

### Sample size and sampling method

The statistical population for this study comprised married individuals who actively used Instagram as their primary social media platform. To determine the sample size, we applied a formula for estimating population proportions, considering a 95% confidence level, a 5% margin of error, and assuming a 50% proportion in the population. The minimum required sample size calculated from this formula was 384. However, in the interest of enhancing result generalizability and accounting for potential missing data, we opted to increase the sample size.

Utilizing a purposive sampling strategy, we aimed to reach a diverse group of married individuals on Instagram. The survey link was strategically disseminated to popular Instagram pages covering a variety of themes, including sports, romance, humor, social issues, and religion. We engaged with page administrators, requesting them to share our survey link on their story pages, thereby targeting a broader audience.

Inclusion criteria for participants were being married, residing in Iran, and actively using Instagram. Due to the fact that the questionnaire was completed electronically, until all the questions were answered, the answers were not recorded, therefore, no exclusion criteria were considered for this study. Our final sample, consisting of 1,756 participants, was selected based on the convenience sampling method. During the sampling process, we designed and hosted a questionnaire on Porsline’s website. The survey link was then shared on Instagram, facilitating easy access for participants. The age range of participants spanned from 18 to 65 years, and the sample exhibited a diverse array of characteristics, including education, income, ethnicity, and religiosity, as detailed in Table 1.

### Materials

The online questionnaire consisted of four sections: demographic and background information, social media and entertainment preferences, sexual satisfaction, and attitude toward marital infidelity.

### Demographic and background information

This section asked the participants to provide information about their age, gender, marital duration, marriage age, spouse’s age, spouse’s education level, spouse’s income level, place of residence, ethnicity, religiosity, and frequency of sexual intercourse.

### Social media and entertainment preferences

This section measured the participants’ choices and frequency of media consumption, such as social networking sites, movies, and series. The participants were asked to indicate how often they used or watched different types of media on a 3-point Likert scale ranging from 1 (less than 1 h) to 3 (more than 2 h). They were also asked to indicate their favorite genres and origins of movies and series on a checklist of options. The social media and entertainment preferences checklist was developed by the researchers based on a review of the literature and a pilot study. The checklist had acceptable reliability and validity, as indicated by a Cronbach’s alpha of 0.82 and a content validity index of 0.91.

### Sexual satisfaction

The inventory of sexual satisfaction was designed by Hudson et al. in 1981 [15]. Answers to the 25 items of this tool are scored based on a 5-point Likert scale. The minimum and maximum scores are 25 and 125 respectively. The validity and reliability of the tool in Iran were confirmed by Bahrami et al [16]. in 2016. Cronbach’s alpha for positive sexual satisfaction questions was 0.80 and the reliability of negative sexual satisfaction questions was 0.77, and the intra-cluster correlation coefficient was calculated to be 0.80. For the measurement of reliability in the present study, Cronbach’s alpha coefficient of sexual satisfaction was calculated to be 0.781.

### Attitude toward marital infidelity

This section measured the participants’ degree of approval or acceptance of extramarital sexual relationships using the Attitudes Toward Infidelity Scale (ATIS) [14]. The ATIS is a 20-item scale that assesses the cognitive and affective aspects of attitude toward marital infidelity. The participants were asked to indicate their agreement or disagreement with each statement on a 5-point Likert scale ranging from 1 (strongly disagree) to 5 (strongly agree). The total score ranged from 20 to 100, with higher scores indicating higher attitude toward marital infidelity. The ATIS has been shown to have good reliability and validity across different cultures and populations [13]. Procedure. The procedure section describes how the study was conducted and how the data was collected and analyzed.

### Statistical analysis

The data was analyzed using SPSS software version 27.0 (SPSS Inc., Chicago, IL, USA). The normality of continuous variables was tested using the Kolmogorov-Smirnov test, histogram, and P-P diagram, which showed that they were not normally distributed. Descriptive statistics were computed for all variables. The groups were compared using the Chi-square test and Mann-Whitney and Kruskal-Wallis non-parametric tests. Generalized Linear Models (GLM) were used to assess the association among sexual satisfaction, demographic variables, use of social media, and attitude towards marital infidelity. To avoid collinearity, we ran two separate GLM models. We reported the regression coefficients (β) and adjusted β (β*) with 95% CI and *P*-value.

## Results

The results of the study are presented in four tables. Table 1 shows the descriptive statistics and the results of the Mann-Whitney U and Kruskal-Wallis H tests for the two outcome variables: sexual satisfaction and attitude toward marital infidelity. Table 2 shows the descriptive statistics and the results of the Mann-Whitney U and Kruskal-Wallis H tests for various variables related to social media and entertainment preferences. Table 3 shows the results of the GIM analysis for sexual satisfaction as the dependent variable and various demographic and social factors as the independent variables. Table 4 shows the results of the GLM analysis for attitude toward marital infidelity as the dependent variable and various factors related to entertainment preferences and sexual satisfaction as the independent variables.

### Sexual satisfaction and attitude toward marital infidelity

Table 1 summarizes the mean scores and standard deviations of sexual satisfaction and attitude toward marital infidelity for different groups based on various demographic and social factors. The results of the non-parametric tests indicate that there are significant differences in sexual satisfaction based on age, spouse’s age, duration of marriage, spouse’s education, and city of residence. There are also significant differences in attitude toward marital infidelity based on gender, age, ethnicity, income level, spouse’s income level, and Iranian social media usage.

The mean score of sexual satisfaction was higher for participants who were less than 30 years old or whose spouses were less than 30 years old than for those who were older or whose spouses were older. The mean score of sexual satisfaction was also higher for participants who had been married for less than 2 years than for those who had been married longer. Participants who had spouses with higher education levels had higher sexual satisfaction than those who had spouses with lower education levels. Participants who lived in other cities than Tehran had higher sexual satisfaction than those who lived in Tehran.

The mean score of attitudes toward marital infidelity was higher for male participants than for female participants. The mean score of attitude toward marital infidelity was also higher for participants who were younger, who belonged to certain ethnicities (such as Indian, Turkish, or Korean), who had higher income levels or whose spouses had lower income levels, and who used Iranian social media more often.

**Table 1:** Comparing the mean of sexual satisfaction and attitudes toward marital infidelity with demographic variables

				Sexual Satisfaction		Attitude Toward Marital Infidelity	
Variables		Frequency	Percent	M ± SD	*P*	M ± SD	*P*
**Gender**	Female	1160	66.1	72.16(5.90)	0.190	35.42(3.20)	**0.001**
	Male	596	33.9	72.57(6.57)		35.95(4.07)	
**Age**	less than 30	622	35.4	72.61(6.07)	**0.025**	35.47(3.50)	**0.038**
	31–40	734	41.8	72.38(6.02)		35.55(3.36)	
	41–50	312	17.8	71.93(6.37)		35.76(3.76)	
	more than 51	88	5.0	70.63(6.49)		36.26(4.05)	
**Spouse’s age**	less than 30	455	25.9	73.10(6.22)	**0.003**	35.75(3.79)	0.124
	31–40	869	49.5	72.20(6.00)		35.41(3.38)	
	41–50	323	18.4	71.61(6.30)		35.78(3.56)	
	more than 51	109	6.2	71.85(6.04)		35.84(3.35)	
**Duration of marriage**	less than 2y	330	18.8	73.69(5.83)	**0.001**	35.06(3.16)	**0.009**
	3-5y	314	17.9	71.85(6.69)		35.93(3.60)	
	6-10y	445	25.3	71.98(6.00)		35.62(3.73)	
	10-20y	468	26.7	72.36(5.92)		35.64(3.42)	
	more than 20y	199	11.3	71.19(6.17)		35.86(3.68)	
**marriage age**	less than 20y	444	25.3	72.47(5.93)	0.261	35.57(3.55)	0.070
	20-25y	663	37.8	72.39(6.04)		35.41(3.49)	
	25-30y	417	23.7	71.79(6.02)		35.64(3.47)	
	more than 30y	232	13.2	72.63(6.92)		36.09(3.61)	
**Age difference with spouse**	less than 1y	332	18.9	72.36(5.97)	0.859	35.59(3.40)	0.138
	2-5y	823	46.9	72.20(6.10)		35.41(3.49)	
	6-10y	475	27.1	72.37(6.33)		35.78(3.49)	
	more than 11y	126	7.2	72.55(6.15)		36.16(4.10)	
**ethnicity**	fars	734	41.8	72.15(6.20)	0.377	35.77(3.51)	**0.010**
	tork	315	17.9	72.55(6.21)		35.26(3.47)	
	lor	164	9.3	72.36(6.33)		35.58(3.60)	
	kord	162	9.2	72.43(5.35)		34.75(3.40)	
	Mazani/Gilak	170	9.7	71.52(6.22)		36.16(3.58)	
	Other(Arab/Torkman/baluch and etc.)	211	12.0	72.94(6.08)		35.68(3.52)	
**BMI**	less than 25	748	42.6	72.30(6.00)	0.942	35.61(3.62)	0.668
	25–30	644	36.7	72.26(6.06)		35.56(3.43)	
	more than 30	289	16.5	72.38(6.65)		35.64(3.46)	
**Religion**	islam(shia)	1533	87.3	72.25(6.17)	0.076	35.65(3.53)	0.237
	islam(soni)	128	7.3	73.39(5.28)		34.94(3.33)	
	other(Atheist and other religions)	95	5.4	71.75(6.48)		35.65(3.52)	
**education**	diploma and below	551	31.4	72.33(6.48)	0.767	35.83(3.64)	0.161
	Master’s and bachelor’s degree	947	53.9	72.31(5.96)		35.42(3.45)	
	Masters and Ph.D	258	14.7	72.19(6.02)		35.75(3.48)	
**Spouse’s Education**	diploma and below	676	38.5	72.14(6.17)	**0.040**	35.65(3.36)	0.251
	Master’s and bachelor’s degree	750	42.7	72.67(5.97)		35.69(3.67)	
	Masters and Ph.D	330	18.8	71.78(6.39)		35.27(3.49)	
**Employee status**	Unemployed	587	33.4	72.14(6.05)	0.535	35.49(3.11)	0.552
	Self employed	717	40.8	72.21(6.27)		35.65(3.79)	
	governmental job	368	21.0	72.67(6.15)		35.74(3.63)	
	Student	84	4.8	72.54(5.51)		35.27(3.41)	
**Income level**	nothing	596	33.9	71.81(5.82)	0.093	35.42(3.11)	**0.001**
	less than 5 m	335	19.1	72.15(6.62)		35.12(3.40)	
	5–10 m	311	17.7	72.79(5.79)		35.36(3.62)	
	10–20 m	347	19.8	72.81(6.31)		36.06(3.85)	
	more than 20 m	167	9.5	72.39(6.42)		36.76(3.97)	
**Spouse’s income**	nothing	330	18.8	72.78(6.39)	0.112	36.04(3.82)	**0.003**
	less than 5 m	341	19.4	72.36(5.97)		35.04(3.47)	
	5–10 m	434	24.7	71.59(6.13)		35.42(3.22)	
	10–20 m	469	26.7	72.42(6.14)		35.73(3.67)	
	more than 20 m	182	10.4	72.72(5.90)		36.00(3.21)	
**city of residence**	Tehran	558	31.8	71.65(6.18)	**0.032**	35.29(3.76)	**0.006**
	other metropolis	491	28.0	72.69(6.08)		36.08(3.39)	
	other cities	707	40.3	72.55(6.10)		35.50(3.38)	

### Social media and entertainment preferences

Table 2 summarizes the mean scores and standard deviations of sexual satisfaction and attitude toward marital infidelity for different groups based on various variables related to social media and entertainment preferences. The results of the non-parametric tests indicate that there are significant differences in sexual satisfaction based on the most number of movies and series watched and the genres of movies and series preferred. There are also significant differences in attitude toward marital infidelity based on almost all the variables except for Skype usage and the genres of movies and series preferred.

The mean score of sexual satisfaction was lower for participants who watched more Iranian or American movies and series than for those who watched other movies and series. The mean score of sexual satisfaction was also higher for participants who preferred to watch comedy, romantic, or action movies and series than for those who preferred other genres.

The mean score of attitude toward marital infidelity was higher for participants who used WhatsApp, Telegram, Instagram, Facebook, YouTube, Snapchat, or Iranian social media more often than for those who used them less often. The mean score of attitude toward marital infidelity was also higher for participants who watched more Indian, American, Turkish, Korean, or other movies and series than for those who watched more Iranian movies and series. Participants who watched sex or porn videos more often had higher attitude toward marital infidelity than those who watched them less often.

**Table 2:** Comparing the mean of sexual satisfaction and attitudes toward marital infidelity with use of Social media and entertainment preferences

				Sexual Satisfaction		Attitude Toward Marital Infidelity	
Variables		Frequency	Percent	M ± SD	*P*	M ± SD	*P*
**For how many hours in a day and night do you use WhatsApp?**	less than 1 h	960	54.7	72.40(6.03)	0.489	35.41(3.36)	**< 0.001**
	1–2 h	317	18.1	72.42(6.21)		35.41(3.39)	
	more than 2 h	479	27.3	72.00(6.30)		36.11(3.88)	
**Telegram**	less than 1 h	1257	71.6	72.37(6.07)	0.173	35.32(3.40)	**< 0.001**
	1–2 h	231	13.2	71.52(6.27)		35.49(3.58)	
	more than 2 h	268	15.3	72.67(6.31)		37.05(3.69)	
**Instagram**	less than 1 h	333	19.0	71.64(6.35)	0.069	35.25(3.40)	**< 0.001**
	1–2 h	414	23.6	72.83(6.10)		35.21(3.32)	
	more than 2 h	1009	57.5	72.30(6.06)		35.88(3.62)	
**Facebook**	less than 1 h	1687	96.1	72.26(6.14)	0.363	35.54(3.47)	**0.002**
	1–2 h	38	2.2	72.81(6.61)		36.92(4.21)	
	more than 2 h	31	1.8	73.62(5.38)		37.03(4.60)	
**clubhouse**	less than 1 h	1720	97.9	72.25(6.12)	0.067	35.57(3.50)	0.078
	1–2 h	22	1.3	74.04(7.24)		37.19(4.65)	
	more than 2 h	14	0.8	75.15(4.98)		36.00(4.39)	
**YouTube**	less than 1 h	1567	89.2	72.28(6.08)	0.983	35.36(3.37)	**< 0.001**
	1–2 h	70	4.0	72.51(5.77)		36.57(4.41)	
	more than 2 h	119	6.8	72.50(7.11)		38.51(3.71)	
**Snapchat**	less than 1 h	1693	96.4	72.27(6.13)	0.503	35.53(3.46)	**< 0.001**
	1–2 h	32	1.8	74.07(7.31)		38.29(3.75)	
	more than 2 h	31	1.8	72.52(4.64)		36.88(5.33)	
**Skype**	less than 1 h	1708	97.3	72.30(6.12)	0.864	35.56(3.48)	**0.013**
	1–2 h	29	1.7	72.30(8.49)		38.03(4.21)	
	more than 2 h	19	1.1	72.50(3.40)		35.68(4.74)	
**Iranian social media**	less than 1 h	1572	89.5	72.30(6.11)	0.074	35.44(3.41)	**< 0.001**
	1–2 h	103	5.9	73.40(6.05)		37.00(4.05)	
	more than 2 h	81	4.6	71.06(6.54)		37.01(4.18)	
**The most number of movies and series you have watched?**	Iranian	930	53.0	72.05(6.07)	0.**033**	35.31(3.39)	**< 0.001**
	Indian(Bollywood)	36	2.1	72.15(8.74)		37.60(3.76)	
	American(Hollywood)	388	22.1	72.12(5.70)		35.63(3.62)	
	Turkish	232	13.2	72.94(6.46)		36.36(3.64)	
	Korean	35	2.0	71.53(5.66)		36.78(3.33)	
	other	135	7.7	73.65(6.42)		35.42(3.52)	
**What genres of movies and series do you prefer to watch?**	drama	105	6.0	71.59(6.54)	**0.001**	35.68(3.84)	0.426
	comedy	345	19.6	72.76(6.25)		35.52(3.32)	
	romantic	371	21.1	73.03(6.54)		35.65(3.52)	
	action	97	5.5	73.54(5.80)		35.74(3.55)	
	family	259	14.7	71.31(6.05)		35.05(3.72)	
	other	552	31.4	71.93(5.71)		35.82(3.47)	
**How often do you watch sex or porn videos in a week?**	less than 1 h	1455	82.9	72.21(6.05)	0.074	35.32(3.27)	**< 0.001**
	1–2 h	112	6.4	72.50(6.56)		36.09(4.30)	
	more than 2 h	181	10.3	72.84(6.68)		37.57(4.23)	

### Generalized linear model

Table 3 shows the results of the GLM analysis for sexual satisfaction as the dependent variable and various demographic and social factors as the independent variables. The results indicate that duration of marriage, ethnicity, spouse’s education, Iranian social media usage, and attitude toward marital infidelity have significant effects on sexual satisfaction in both the univariable and multivariable analyses.

Participants who had been married for less time had higher sexual satisfaction than those who had been married longer. Participants who belonged to the Mazani/Gilak ethnicity had lower sexual satisfaction than those who belonged to other ethnicities. Participants who had spouses with higher education levels had higher sexual satisfaction than those who had spouses with lower education levels. Participants who used Iranian social media more often had higher sexual satisfaction than those who used it less often. Participants who had higher attitude toward marital infidelity also had higher sexual satisfaction.

**Table 3:** The univariable and multivariable generalized linear models (GLM) to determine the association among sexual satisfaction and demographic variables, use of social media, and attitude towards marital infidelity

Dependent variable: Sexual satisfaction		Univariable		Multivariable	
**Variables**		β (95% CI)	*P*-value	β (95% CI)	*P*-value
**Gender**	Female	− 0.415(-1.03-0.20)	0.190	-	-
	Male	Reference	-	-	-
**Age**	less than 30	1.97(0.57-3.38)	0.006*	-	-
	31–40	1.75(0.35-3.14)	0.014*	-	-
	41–50	1.30(-0.18-2.79)	0.087	-	-
	more than 51	Reference	-	-	-
**Spouse’s age**	less than 30	1.24(-0.06-2.55)	0.062	-	-
	31–40	0.34(-0.90-1.59)	0.587	-	-
	41–50	− 0.23(-1.59-1.11)	0.730	-	-
	more than 51	Reference	-	-	-
**Duration of marriage**	less than 2y	2.49(1.40–3.58)	0.000*	2.491(0.59-4.38)	0.010**
	3-5y	0.65(-0.44-1.76)	0.243	0.57(-1.23-2.38)	0.534
	6-10y	0.78(-0.25-1.82)	0.138	0.68(-0.87-2.24)	0.389
	10-20y	1.164(0.13-2.19)	0.027*	1.02(-0.27-2.31)	0.124
	more than 20y	Reference	-	Reference	-
**marriage age**	less than 20y	− 0.16(-1.15-0.83)	0.751	-	-
	20-25y	− 0.24(-1.18-0.69)	0.608	-	-
	25-30y	− 0.84(-1.84-0.16)	0.102	-	-
	more than 30y	Reference	-	-	-
**Age difference with spouse**	less than 1y	− 0.19(-1.48-1.10)	0.771	-	-
	2-5y	− 0.35(-1.54-0.82)	0.553	-	-
	6-10y	− 0.18(-1.42-1.05)	0.771	-	-
	more than 11y	Reference	-	-	-
**ethnicity**	Fars	− 0.79(-1.76-0.17)	0.108	− 0.61(-1.59-0.35)	0.215
	Tork	− 0.38(-1.48-0.70)	0.488	− 0.21(-1.32-0.88)	0.700
	Lor	− 0.57(-1.85-0.69)	0.375	− 0.46(-1.73-0.80)	0.470
	Kord	− 0.51(-1.82-0.80)	0.447	− 0.80(-2.18-0.57)	0.251
	Mazani/Gilak	-1.41(-2.69–0.14)	0.030*	-1.53(-2.84–0.23)	0.021**
	Other(Arab/Torkman/Baluch and etc.)	Reference	-	Reference	-
**BMI**	less than 25	− 0.08(-0.91-0.75)	0.850	-	-
	25–30	− 0.11(-0.96-0.73)	0.791	-	-
	more than 30	Reference	-	-	-
**Religion**	islam(shia)	0.50(-0.78-1.79)	0.446	-	-
	islam(soni)	1.64(-0.03-3.31)	0.054	-	-
	other(atheist/other religions)	Reference	-	-	-
**education**	diploma and below	0.13(-0.78-1.05)	0.772	-	-
	Master’s and bachelor’s degree	0.12(-0.73-0.98)	0.776	-	-
	Masters and Ph.D.	Reference	-	-	-
**Spouse’s Education**	diploma and below	0.36(-0.46-1.18)	0.390	-	-
	Master’s and bachelor’s degree	0.89(0.08-1.70)	0.031*	-	-
	Masters and Ph.D.	Reference	-	-	-
**Employee status**	Unemployed	− 0.39(-1.84-1.04)	0.591	-	-
	Self employed	− 0.33(-1.76-1.09)	0.650	-	-
	governmental job	0.13(-1.36-1.62)	0.864	-	-
	Student	Reference	-	-	-
**Income level**	nothing	− 0.57(-1.67-0.52)	0.307	-	-
	less than 5 m	− 0.23(-1.41-0.94)	0.698	-	-
	5–10 m	0.40(-0.79 − 1.60)	0.506	-	-
	10–20 m	0.42(-0.76 − 1.60)	0.486	-	-
	more than 20 m	Reference	-	-	-
**Spouse’s income**	nothing	0.06(-2.22–0.03)	0.916	-	-
	less than 5 m	− 0.36(-1.49-0.77)	0.531	-	-
	5–10 m	-1.12(-2.22–0.03)	0.044*	-	-
	10–20 m	− 0.30(-1.38-0.78)	0.586	-	-
	more than 20 m	Reference	-	-	-
**city of residence**	Tehran	− 0.89(-1.58–0.19)	0.012*	-	-
	other metropolis	0.144(-0.57-0.86)	0.694	-	-
	other cities	Reference	-	-	-
**For how many hours in a day and night do you use WhatsApp?**	less than 1 h	0.39(-0.29-1.08)	0.259	-	-
	1–2 h	0.41(-0.47-1.31)	0.358	-	-
	more than 2 h	Reference	-	-	-
**Telegram**	less than 1 h	− 0.30(-1.14-0.53)	0.480	-	-
	1–2 h	-1.15(-2.26–0.04)	0.041	-	-
	more than 2 h	Reference	-	-	-
**Instagram**	less than 1 h	− 0.65(-1.42-0.11)	0.093	-	-
	1–2 h	0.53(-0.18-1.24)	0.147	-	-
	more than 2 h	Reference	-	-	-
**Facebook**	less than 1 h	-1.35(-3.60-0.90)	0.239	-	-
	1–2 h	− 0.80(-3.77-2.16)	0.595	-	-
	more than 2 h	Reference	-	-	-
**clubhouse**	less than 1 h	-2.89(-6.24-0.45)	0.090	-	-
	1–2 h	-1.10(-5.34-3.13)	0.609	-	-
	more than 2 h	Reference	-	-	-
**YouTube**	less than 1 h	− 0.22(-1.46-1.00)	0.715	-	-
	1–2 h	0.00(-1.89-1.90)	0.996	-	-
	more than 2 h	Reference	-	-	-
**Snapchat**	less than 1 h	− 0.24(-2.67-2.17)	0.841	-	-
	1–2 h	1.55(-1.78-4.89)	0.361	-	-
	more than 2 h	Reference	-	-	-
**Skype**	less than 1 h	− 0.19(-3.22-2.82)	0.898	-	-
	1–2 h	− 0.19(-4.01-3.63)	0.921	-	-
	more than 2 h	Reference	-	-	-
**Iranian social media**	less than 1 h	1.23(-0.14-2.62)	0.080	1.33(-0.14 − 2.80)	0.078
	1–2 h	2.33(0.49-4.18)	0.013*	1.93(0.10-3.76)	0.039**
	more than 2 h	Reference	-	Reference	-
**The most number of movies and series you have watched?**	Iranian	-1.59(-2.70–0.48)	0.005*	-	-
	Indian(Bollywood)	-1.50(-3.82-0.82)	0.207	-	-
	American(Hollywood)	-1.52(-2.73–0.32)	0.013*	-	-
	Turkish	− 0.70(-2.02-0.61)	0.296	-	-
	Korean	-2.12(-4.47-0.23)	0.078	-	-
	other	Reference	-	-	-
**What genres of movies and series do you prefer to watch?**	drama	− 0.34(-1.64-0.95)	0.605	-	-
	comedy	0.83(0.00-1.66)	0.050	-	-
	romantic	1.10(0.29-1.91)	0.008*	-	-
	action	1.61(0.25-2.97)	0.020*	-	-
	family	− 0.62(-1.53-0.28)	0.180	-	-
	other	Reference	-	-	-
**How often do you watch sex or porn videos in a week?**	less than 1 h	− 0.62(-1.61-0.35)	0.211	-	-
	1–2 h	− 0.33(-1.87-1.19)	0.665	-	-
	more than 2 h	Reference	-	-	-
**Attitude towards marital infidelity**		0.16(0.08-0.25)	0.000*	0.17(0.08-0.25)	0.000**

Table 4 shows the results of the GLM analysis for attitude toward marital infidelity as the dependent variable and various factors related to entertainment preferences and sexual satisfaction as the independent variables. The results indicate that duration of marriage, marriage age, spouse’s education, Telegram usage, YouTube usage, Iranian social media usage, how often sex or porn videos are watched, and sexual satisfaction have significant effects on attitude toward marital infidelity in both the univariable and multivariable analyses.

Participants who had been married for less time or who got married at a younger age had higher attitude toward marital infidelity than those who had been married longer or who got married at an older age. Participants who had spouses with higher education levels had higher attitude toward marital infidelity than those who had spouses with lower education levels. Participants who used Telegram, YouTube, or Iranian social media more often had higher attitude toward marital infidelity than those who used them less often. Participants who watched sex or porn videos more often also had higher attitude toward marital infidelity than those who watched them less often. Participants who had higher sexual satisfaction also had higher attitude toward marital infidelity.

**Table 4:** The univariable and multivariable generalized linear models (GLM) to determine the association among attitude towards marital infidelity and demographic variables, use of social media and sexual satisfaction

Dependent variable: Attitude towards marital infidelity		Univariable		Multivariable	
**Variables**		β (95% CI)	*P*-value	β (95% CI)	*P*-value
**Gender**	Female	− 0.53(-0.89–0.18)	0.003*	-	-
	Male	Reference	-	-	-
**Age**	less than 30	− 0.789(-1.59-0.02)	0.056	-	-
	31–40	− 0.70(-1.50-0.09)	0.084	-	-
	41–50	− 0.49(-1.35-0.36)	0.256	-	-
	more than 51	Reference	-	-	-
**Spouse’s age**	less than 30	− 0.08(-0.84-0.66)	0.820	-	-
	31–40	− 0.42(-1.14-0.29)	0.243	-	-
	41–50	− 0.06(-0.84-0.72)	0.880	-	-
	more than 51	Reference	-	-	-
**Duration of marriage**	less than 2y	− 0.80(-1.43–0.17)	0.012*	-1.16(-2.17–0.15)	0.024**
	3-5y	0.06(-0.56-0.70)	0.833	− 0.34(-1.31-0.61)	0.480
	6-10y	− 0.24(-0.84-0.35)	0.427	− 0.48(-1.31-0.34)	0.248
	10-20y	− 0.22(-0.81-0.37)	0.463	− 0.12(-0.81-0.56)	0.717
	more than 20y	Reference	-	Reference	-
**marriage age**	less than 20y	− 0.52(-1.09-0.05)	0.074	− 0.82(-1.62–0.02)	0.043**
	20-25y	− 0.68(-1.22–0.14)	0.012*	− 0.72(-1.40–0.04)	0.036**
	25-30y	− 0.45(-1.02-0.12)	0.126	− 0.35(-0.96-0.26)	0.261
	more than 30y	Reference	-	Reference	-
**Age difference with spouse**	less than 1y	− 0.56(-1.30-0.17)	0.136	-	-
	2-5y	− 0.74(-1.42–0.06)	0.032*	-	-
	6-10y	− 0.37(-1.08-0.33)	0.300	-	-
	more than 11y	Reference	-	-	-
**ethnicity**	Fars	0.09(-0.46-0.64)	0.750	-	
	Tork	− 0.42(-1.04-0.20)	0.189	-	-
	Lor	− 0.10(-0.83-0.62)	0.785	-	-
	Kord	− 0.93(-1.68–0.18)	0.015*	-	-
	Mazani/Gilak	0.47(-0.25 − 1.20)	0.201	-	-
	Other(Arab/Torkman/Baluch and etc.)	Reference	-	-	-
**BMI**	less than 25	− 0.02(-0.50-0.45)	0.931	-	-
	25–30	− 0.07(-0.56-0.41)	0.759	-	-
	more than 30	Reference	-	-	-
**Religion**	islam(shia)	− 0.00(-0.74-0.74)	0.996	-	-
	islam(soni)	− 0.70(-1.66-0.25)	0.152	-	-
	other(atheist/other religions)	Reference	-	-	-
**education**	diploma and below	0.08(-0.44-0.61)	0.762	-	-
	Master’s and bachelor’s degree	− 0.32(-0.81-0.16)	0.194	-	-
	Masters and Ph.D.	Reference	-	-	-
**Spouse’s Education**	diploma and below	0.37(-0.10-0.84)	0.122	0.69(0.17 − 1.20)	0.009**
	Master’s and bachelor’s degree	0.41(-0.04-0.88)	0.078	0.54(0.08 − 1.00)	0.020**
	Masters and Ph.D.	Reference	-	Reference	-
**Employee status**	Unemployed	0.21(-0.61-1.04)	0.617	-	-
	Self employed	0.37(-0.44-1.19)	0.367	-	-
	governmental job	0.46(-0.39-1.32)	0.286	-	-
	Student	Reference	-	-	-
**Income level**	nothing	-1.33(-1.96–0.70)	0.000*	-	-
	less than 5 m	-1.63(-2.30–0.95)	0.000*	-	-
	5–10 m	-1.39(-2.07–0.71)	0.000*	-	-
	10–20 m	− 0.70(-1.37–0.02)	0.042*	-	-
	more than 20 m	Reference	-	-	-
**Spouse’s income**	nothing	0.04(-0.61-0.70)	0.893	-	-
	less than 5 m	− 0.95(-1.60–0.30)	0.004*	-	-
	5–10 m	− 0.58(-1.20-0.04)	0.070	-	-
	10–20 m	− 0.26(-0.88-0.36)	0.410	-	-
	more than 20 m	Reference	-	-	-
**city of residence**	Tehran	− 0.21(-0.61-0.18)	0.301	-	-
	other metropolis	0.57(0.16-0.99)	0.006*	-	-
	other cities	Reference	-	-	-
**For how many hours in a day and night do you use WhatsApp?**	less than 1 h	− 0.69(-1.09–0.30)	0.001*	-	-
	1–2 h	− 0.69(-1.20–0.18)	0.008*	-	-
	more than 2 h	Reference	-	-	-
**Telegram**	less than 1 h	-1.73(-2.20–1.25)	0.000*	− 0.69(-1.23–0.15)	0.012**
	1–2 h	-1.56(-2.19–0.93)	0.000*	− 0.66(-1.32–0.01)	0.045**
	more than 2 h	Reference	-	Reference	-
**Instagram**	less than 1 h	− 0.62(-1.06–0.18)	0.006*	-	-
	1–2 h	− 0.67(-1.08–0.25)	0.001*	-	-
	more than 2 h	Reference	-	-	-
**Facebook**	less than 1 h	-1.49(-2.78–0.19)	0.024*	-	-
	1–2 h	− 0.11(-1.81-1.58)	0.896	-	-
	more than 2 h	Reference	-	-	-
**clubhouse**	less than 1 h	− 0.42(-2.34-1.49)	0.667	-	-
	1–2 h	1.19(-1.24-3.62)	0.338	-	-
	more than 2 h	Reference	-	-	-
**YouTube**	less than 1 h	-3.15(-3.84–2.46)	0.000*	-1.52(-2.38–0.67)	0.000**
	1–2 h	-1.94(-3.00–0.87)	0.000*	− 0.97(-2.09-0.13)	0.086
	more than 2 h	Reference	-	Reference	-
**Snapchat**	less than 1 h	-1.34(-2.72-0.04)	0.057	-	-
	1–2 h	1.41(-0.49-3.32)	0.145	-	-
	more than 2 h	Reference	-	-	-
**Skype**	less than 1 h	− 0.12(-1.85-1.60)	0.887	-	-
	1–2 h	2.35(0.16-4.53)	0.035*	-	-
	more than 2 h	Reference	-	-	-
**Iranian social media**	less than 1 h	-1.57(-2.36–0.78)	0.000*	-	-
	1–2 h	− 0.01(-1.06-1.03)	0.981	-	-
	more than 2 h	Reference	-	-	-
**The most number of movies and series you have watched?**	Iranian	− 0.11(-0.74-0.51)	0.725	0.30(-0.34-0.94)	0.358
	Indian(Bollywood)	2.17(0.84 − 3.50)	0.001*	2.14(0.84-3.43)	0.001**
	American(Hollywood)	0.20(-0.48-0.89)	0.554	0.13(-0.55-0.82)	0.698
	Turkish	0.93(0.18-1.69)	0.015*	0.74(-0.01-1.49)	0.056
	Korean	1.35(0.00-2.69)	0.049*	1.01(-0.33-2.36)	0.139
	other	Reference	-	Reference	-
**What genres of movies and series do you prefer to watch?**	drama	− 0.14(-0.89-0.60)	0.713	− 0.34(-1.05-0.37)	0.352
	comedy	− 0.29(-0.77-0.18)	0.229	− 0.26(-0.75-0.22)	0.285
	romantic	− 0.16(-0.63-0.30)	0.487	− 0.37(-0.86-0.10)	0.126
	action	− 0.07(-0.86-0.70)	0.849	− 0.23(-0.99-0.52)	0.549
	family	− 0.76(-1.28–0.23)	0.004*	− 0.54(-1.08–0.00)	0.049**
	other	Reference	-	Reference	-
**How often do you watch sex or porn videos in a week?**	less than 1 h	-2.25(-2.80–1.70)	0.000*	− 0.77(-1.53–0.00)	0.048**
	1–2 h	-1.48(-2.34–0.62)	0.001*	− 0.44(-1.36-0.46)	0.338
	more than 2 h	Reference	-	Reference	-
**Sexual satisfaction**		0.05(0.02-0.08)	0.000*	0.05(0.02-0.08)	0.000**

## Discussion

The aim of this study was to investigate the relationship between social media and entertainment preferences and sexual satisfaction and attitude toward marital infidelity among married couples in Iran. The study used a cross-sectional design and collected data from 1756 married participants using an online questionnaire. The study employed descriptive statistics, non-parametric tests, and GLM to analyze the data.

In our discussion, we acknowledge the nuanced differences observed between our study and prior literature, emphasizing the influence of participant demographics and cultural contexts. Notably, our findings diverge from those of a U.S.-based study [17], which reported higher sexual satisfaction among older couples with longer marriages, higher income, and education levels. Our results, conducted in Iran, indicate higher sexual satisfaction among younger couples, those with shorter marriages, higher spouse education, and residence outside Tehran. These disparities suggest cultural and social factors, possibly linked to differing norms and expectations surrounding sexuality and marriage in Iranian and American cultures. For instance, Iranian cultural values, such as youthfulness and education, may play a more pivotal role in shaping sexual satisfaction. Additionally, our findings on attitudes toward marital infidelity, contrasting with another Iranian study [18], underscore the impact of individual and relational factors, potentially influenced by unique Iranian cultural dynamics, such as gender inequality, age diversity, income disparity, urbanization, and heightened social media exposure. Consequently, our study contributes fresh insights into the complex interplay between sexual satisfaction, attitudes toward marital infidelity, and cultural nuances among married couples in Iran, offering implications for counseling, gender equality promotion, and social media regulation. While recognizing limitations, such as sample size and the need for diverse measures, our study sets the stage for future research to delve deeper into these intricate dynamics across varied cultural landscapes.

However, the study also revealed some novel and interesting findings that have not been widely explored in the existing literature. For instance, the study found that sexual satisfaction was lower among participants who watched more Iranian or American movies and series and higher among participants who watched more comedy, romantic, or action movies and series. These findings suggest that the type and origin of the movies and series that married couples watch may have an impact on their sexual satisfaction, possibly by influencing their expectations, attitudes, or behaviors regarding sexuality. The study also found that attitude toward marital infidelity was higher among participants who watched more Indian, American, Turkish, Korean, or other movies and series and lower among participants who preferred to watch family movies and series. These findings imply that the type and origin of the movies and series that married couples watch may also affect their attitude toward marital infidelity, possibly by influencing their values, norms, or perceptions regarding fidelity. These findings are consistent with the media effects theory, which posits that media consumption can shape or reflect the beliefs, attitudes, or behaviors of individuals or groups [19].

Another noteworthy finding of the study was that sexual satisfaction and attitude toward marital infidelity were positively correlated and mutually reinforcing. This finding indicates that higher sexual satisfaction may increase attitude toward marital infidelity and vice versa. This finding is contrary to the common assumption that higher sexual satisfaction may reduce attitude toward marital infidelity by enhancing marital quality or satisfaction [20–22]. This finding may be explained by the social exchange theory, which proposes that individuals evaluate their relationships based on the costs and benefits they perceive [23]. According to this theory, higher sexual satisfaction may increase the perceived benefits of the relationship, but also increase the perceived costs of losing the relationship. This may lead to a higher attitude toward marital infidelity as a way of reducing the perceived costs or increasing the perceived benefits of alternative relationships [23, 24].

The findings of this study also have implications for understanding the relationship between mental and sexual health in married people. Previous research has shown that sexual satisfaction and attitude toward marital infidelity can affect and be affected by various mental health factors, such as mood, anxiety, stress, self-esteem, and personality [25–33]. This study adds to this literature by demonstrating that social media and entertainment preferences can also influence sexual satisfaction and attitude toward marital infidelity, possibly by mediating or moderating the effects of mental health factors. For example, social media and entertainment preferences may reflect or shape the values, norms, expectations, and behaviors of married couples regarding sexuality and fidelity, which may in turn affect their mental well-being and their relationship quality. Alternatively, social media and entertainment preferences may act as coping mechanisms or sources of support for married couples who experience mental health challenges or relationship difficulties, which may in turn affect their sexual satisfaction and attitude toward marital infidelity. These possible pathways need to be further explored in future research.

The findings of this study have several implications for theory and practice. First, they contribute to the existing literature on the factors that influence sexual satisfaction and attitude toward marital infidelity among married couples by highlighting the role of social media and entertainment preferences as potential predictors or moderators of these outcomes. Second, they suggest that interventions that aim to enhance sexual satisfaction or prevent marital infidelity among married couples should consider not only individual or relational factors, but also social and cultural factors such as social media and entertainment preferences. Third, they indicate that clinicians who work with married couples should assess their social media and entertainment preferences as well as their sexual satisfaction and attitude toward marital infidelity as part of their diagnosis or treatment plan. They should also provide psychoeducation or counseling about the possible impact of social media and entertainment preferences on their sexual satisfaction and attitude toward marital infidelity and help them develop healthy and balanced habits regarding their social media and entertainment consumption as well as their sexual communication and intimacy.

However, this study also has some limitations that need to be acknowledged. One limitation is the cross-sectional design of the study, which does not allow for causal inferences or temporal changes. A longitudinal design would be more suitable to examine the causal relationships and the dynamics of sexual satisfaction and attitude toward marital infidelity over time. Another limitation is the self-report nature of the data collection method, which may introduce biases such as social desirability or recall errors. A more objective or triangulated method would be more reliable to measure these sensitive variables. A third limitation is the convenience sampling method used to recruit participants online, which may limit the generalizability of the findings to other populations or contexts. A more representative or random sampling method would be more valid to generalize the findings to the broader population of married couples in Iran.

Therefore, future research should address these limitations by using a longitudinal design, a more objective or triangulated data collection method, and a more representative or random sampling method. Additionally, future research could explore other factors that may influence sexual satisfaction and attitude toward marital infidelity among married couples in Iran or other countries with different cultural backgrounds. Furthermore, future research could test the effectiveness of interventions that target social media and entertainment preferences as well as individual or relational factors to improve sexual satisfaction or prevent marital infidelity among married couples.

## Conclusion

In conclusion, this study showed that social media and entertainment preferences are important factors that influence sexual satisfaction and attitude toward marital infidelity among married couples in Iran. The study also suggested some directions for future research on this topic. By understanding how social media and entertainment preferences affect sexual health and marital quality, we can better promote healthy and happy marriages in the modern era.

## Data Availability

The datasets used and/or analyzed during the current study are available from the corresponding author on reasonable request.
